# Juvenile Crime

**Published:** 1896-07-04

**Authors:** 


### Modern Sociology.

### JUVENILE CRIME.

The first part of the Blue Book containing the statistics of crime for 1894 has recently been presented to the House of Commons. It relates to the criminal proceedings, police, coroners, prisons, reformatory and industrial schools, and criminal lunatics, during that period, and demands careful study on the part of sociologists. A comparison of the figures with those for 1893 shows that there is a slight decrease in the number of persons sent for trial at Assizes and Quarter Sessions, and this extends to each of the six classes of crime into which the report is divided. This is so far satisfactory, and it is well known that of recent years there has been a marked diminution in crime. The decrease noted this year, however, is below the average, and the question arises whether there is now a check in the decrease of crime, and whether the average tends to become stationary. Take, by way of example, one class of crime, that of offences against the person. In 1893 there were registered 2,708 offences of this nature; in 1894 2,660, a decrease of 48. Under the head of miscellaneous transgressions there is also a net decrease, though a small one; but one or two items, viz., keeping disorderly houses and attempted suicides, have increased. The decrease all round appears less than formerly, and amongst the professional criminal classes, that is, those who habitually rely upon crime as a means of livelihood, the tendency of crime to diminish seems exhausted. It does not, however, follow that because there is an increase in the number of crimes committed that there is of necessity any corresponding increase in the number of criminals. Sentences are shorter than formerly, and the tendency to mitigate the severity of punishments allows of more criminals being at large at one time. During the ten years 1883—93 the advance in the number of more lenient sentences has been very noticeable. Thus a habitual criminal, who would previously have been sentenced twice in ten years, may now be committed to prison four or five times during the same period. It should also be noted that there has been an increase in the proportion of persons previously convicted, the ratio having risen from 50 to 54·5 per cent. This is doubtless due to the more elaborate system of identification of previously-convicted criminals now in vogue. And this rise in the number of previous convictions known to the police may be expected to continue, as the plan of identifying by anthropometry and finger marks has now been adopted, and will help to swell the number of those whose antecedents are known. There is one feature of the report which, it must be confessed, is very serious, and to which it is the object of this article more especially to direct attention. There is a considerable increase in the number of juvenile offenders especially between the ages of sixteen and twenty-one. The proportion of youthful criminals between these ages has risen from 321 to 330 per 100,000 of the population. This phenomenon is not confined to England alone, but is also seen in France, whilst in Germany the number of minors convicted has increased no less than 32 per cent. It is evident that we have not as yet solved the problem of dealing with juvenile offenders. More than one-third of the number of convicted burglars in 1894 were youths between the ages of sixteen and twenty-one; and, although not all of these join the ranks of the professional criminal classes in after life, yet it must clearly be from this source that their numbers are recruited. This growth in the number of juvenile criminals is the more serious because it is contrary to the tendency observed during many years past. For the last twenty or thirty years the proportion of offenders under sixteen to the total prison population has remarkably decreased. During the first quarter of the century juvenile crime was the despair of the thoughtful, and baffled statesmen. Between 1857 and 1866 the proportion of offenders under sixteen who were committed to prison was 6¾ per cent. In 1891 it had sunk to 2 per cent. Unquestionably this improvement must be traced to the establishment of State elementary, industrial, and reformatory schools. But we have not yet hit upon a satisfactory way of dealing with the youthful offender between the ages of sixteen and twenty-one. These years cover the period of adolescence, a very critical one in the lives of young people of both sexes. Bad habits formed at this time are with difficulty eradicated, whilst, on the other hand, if we could prevent youths from embarking upon a career of crime at this age there would be a great drop in the sum total of criminality. How is the number of these recruits to the criminal classes to be lessened? It has been suggested that a penal reformatory should be established, an institution of a stricter character than the reformatory, for youths on the downward path. An establishment of this description might deal with habitual offenders under the age of twenty-one. The deterrent effects of punishment are smaller upon the young than upon the adult, whilst the young are more susceptible to reformative influences. Again, an increased certainty in the punishment of offences would result in the diminishing of crime. It is not the severity of the punishment, but the certainty with which it will follow the offence that will most deter the young from wrong doing. Uncertainty of punishment encourages crime, but severity may be lessened, provided there is an increase in the probability of detection and punishment. An incalculable amount of mischief is wrought by allowing young people to congregate in knots at the street corners. Loafing becomes a habit, and loafing leads to bad company, and that to crime. Probably more harm is picked up by boys and girls during the hour or two after dusk than in all the rest of the day. Parents of the lower and middle classes are far too careless in the way in which they permit their children to play in the streets. Something effective might, perhaps, be achieved by the State if the age at which children are permitted to leave school was raised to fourteen years, and by placing the Poor Law schools under the management of the Education Department. But upon the whole we must look to a greater vigilance and watchfulness upon the part of parents if there is to be any great diminution in the statistics of juvenile crime.

